# Donor-site Morbidity after Retromolar Bone Harvesting Using a Standardised Press Fit Cylinder Protocol

**DOI:** 10.3390/ma12223802

**Published:** 2019-11-19

**Authors:** Philipp Streckbein, Mathias Meier, Christopher Kähling, Jan-Falco Wilbrand, Tobias Langguth, Heidrun Schaaf, Hans-Peter Howaldt, Roland Streckbein, Sameh Attia

**Affiliations:** 1 Department of Cranio-Maxillofacial Surgery, Justus-Liebig University Giessen, Klinikstr. 33, 35392 Giessen, Germany; Mathias-Meier@web.de (M.M.); christopher.kaehling@uniklinikum-giessen.de (C.K.); jan-falco.wilbrand@uniklinikum-giessen.de (J.-F.W.); Tobias.Langguth@dentist.med.uni-giessen.de (T.L.); heidrun.schaaf@uniklinikum-giessen.de (H.S.); hp.howaldt@uniklinikum-giessen.de (H.-P.H.); sameh.attia@dentist.med.uni-giessen.de (S.A.); 2 IZI - Institute for Postgraduate Education in Dental Implantology, Auf dem Schafsberg, 65549 Limburg, Germany; r.streckbein@izi-online.de

**Keywords:** alveolar ridge augmentation, bone reconstruction, horizontal bone defect, press fit, autologous bone graft, trephine drill

## Abstract

Precise fitting and immobilisation of bone transplants at the recipient site is of utmost importance for the healing process. With the help of the standardised Osseo Transfer System, the recipient site is adjusted to the graft, rather than vice versa as it is typically done. The aim of this study was to analyse donor-site morbidity after harvesting cylindrical bone grafts from the retromolar region using the Osseo Transfer System. The patient satisfaction with the surgical procedures was also evaluated. All patients treated with this standardised reconstruction method between 2006 and 2013 at the Department of Cranio-Maxillofacial Surgery, University Hospital Giessen, were included in this study. Complications were recorded and evaluated. Bone graft success and patient satisfaction were documented with a questionnaire, and then confirmed by clinical and radiological follow-up examinations. Fifty-four patients were treated and 64 harvested cylindrical autologous bone grafts were transplanted. In all cases, dental implants could be inserted after bone healing. One patient lost an implant, associated with failure of the bone graft. Six patients who were examined continued to show neurological disorders in locally limited areas. No complete or long-term damage of the inferior alveolar nerve occurred. More than 94% (n = 52) of the patients were ‘very satisfied’ or ‘satisfied’ with the results and would recommend this surgical treatment to other patients. The standardised Osseo Transfer was an effective treatment option for small and mid-sized alveolar ridge augmentations. A low donor-site morbidity rate and a high transplant success rate were verified. The Osseo Transfer System demonstrated to be a reliable surgical technique without major complications. We highly recommend this surgical augmentation procedure as a surgical treatment for local bone defects.

## 1. Introduction

The lack of teeth is usually a result of tooth extraction or trauma, but some patients may have dental aplasia [1,2]. Missing teeth for more than three months can lead to a local atrophy of the alveolar ridge due to the lack of chewing forces and a functional stimulus [3,4]. Dental implants can only be inserted if adequate bone volume is available to ensure the primary stability of implants and stable osseointegration [2]. Implants must be inserted into the bone so that the rough surface is completely surrounded by well-perfused bone to allow long-term success without peri-implantitis [5]. Depending on the implant system, a minimum of 6–7 mm mesiodistal and 5–6 mm buccolingual alveolar bone widths are suggested for successful implant placement [6]. Otherwise, alveolar bone augmentation before dental implant insertion is required [2]. Autologous bone grafts (e.g., from the iliac crest) are considered the gold standard for augmentation and used mainly to restore large volumes of alveolar bone atrophy [7]. In small- and medium-sized bone defects, an intraoral bone graft can be used to augment the atrophic areas. Bone can be harvested either from the retromolar area of the mandible, chin, or maxilla [8,9]. Other than autografts, allografts, xenografts, or for some indications a combination of both can be used for bone reconstruction [10]. Autografts are preferably used due to their biocompatibility and osteogenesis properties which lead to better bone regeneration [2,7]. Autologous bone grafts from the retromolar region of the mandible have some significant advantages: they can be harvested in local anaesthesia, the amount of available bone can be easily estimated in a standard panoramic X-ray, and minimal resorption of the mainly compact bone can be expected [9]. 

**Figure 1:**
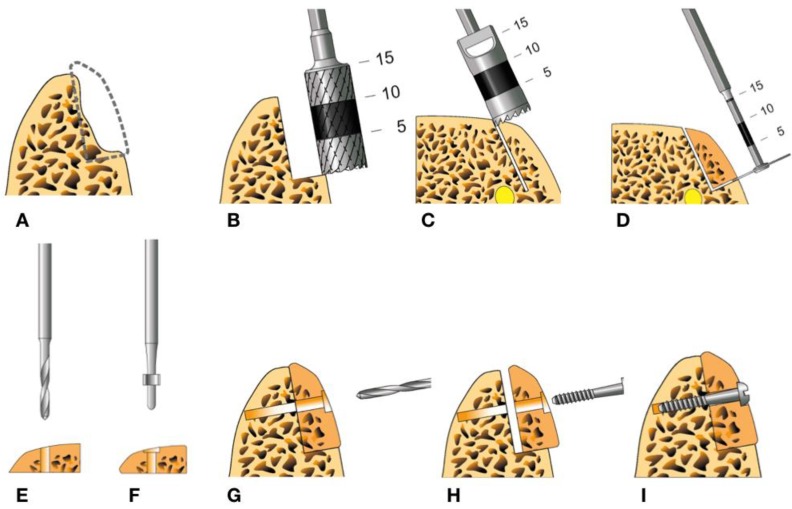
Schematic illustration of the Osseo Transfer System protocol: (**A**) The recipient site. (**B**) Extending the bone defect on the recipient site with a grinding burr to obtain a standardised bone defect. (**C**) Cutting out a bone cylinder from the donor site using a trephine drill to harvest a matching piece of bone. (**D**) Transection the bone cylinder using a micro saw. (**E**) Drilling of a gliding hole in the centre of the bone graft. (**F**) Drilling countersink to avoid extending the screw head outside the graft. (**G**) Adapting the graft to the standardised defect on the recipient’s site and drilling through the graft. (**H**,**I**) Graft fixation using micro-lag-screw.

Bone harvesting can be performed using various instruments, such as drills, chisels, a saw or via piezosurgery [8,9]. With the Osseo Transfer System (BEGO Implant Systems, Bremen, Germany), cylindrical bone grafts can be harvested by using trephines. Three cylindrical burrs with three different diameters are available to harvest a piece of bone that precisely fits to the local defect (Figure 1). This set of instruments was introduced in 2006 as a new augmentation tool for minimally invasive reconstruction of small- and medium-sized jaw defects [11]. The uniqueness of the procedure is that the recipient region can be modulated according to the transplant, not vice versa. The Osseo Transfer System can transform alveolar ridge defects to standardised recipient site defects which enable a perfect geometric matching of the press fitting graft to the defect. Fixation of the graft is performed by using a single lag screw. This leads to high retention and stability of the transplanted bone and maximizes the surface contact to the residual bone for an optimal healing process. When using this standardised procedure, there is no need for manual graft moulding, therefore, the operation time will be reduced [11]. The Osseo Transfer System is mainly used for horizontal augmentation of the alveolar ridge but vertical deficiencies can also be addressed [11]. The standardised procedures have been applied routinely as a minimal invasive surgical procedure in the Department of Cranio-Maxillofacial Surgery, Giessen, Germany. To date, no relevant data concerning complications or transplantation success of this procedure have been published yet.

The aim of this retrospective study was to analyse donor-site complications associated with this procedure. The data obtained are intended to demonstrate the success of the augmentation procedure and patient satisfaction.

## 2. Material and Methods

This retrospective, clinical, observational study includes all patients treated in our Department of Cranio-Maxillofacial Surgery, University Hospital Giessen, Germany, from April 2006 to February 2013, who received a standardised bone graft of the retromolar region by using the Osseo Transfer System. A total of 54 patients with bone defects were augmented with press fit bone cylinder. Fifty-two patients completed the questionnaire and 48 patients accepted the follow up examination. In one patient, bone was harvested from both mandibular angles, hence we count a total of 49 bone donor sites. Surgical protocols recommended by the manufacturer were followed for all transplanted bone. Data were collected from patient charts including age, sex, indication for treatment, defects and bone cylinder size. Information about antibiotic administration, surgery duration and intraoperative complications were also recorded. All included patients were scheduled for follow-up examinations and asked to fill out a customized questionnaire. The main purpose of the assessment was to determine the complications and to describe the rate of donor-site morbidity. Postoperative complications include wound infection, wound dehiscence, secondary haemorrhage, haematoma, pain, nerve injury and limitations of speech and swallowing. Duration and intensity of the complications were also specified. Furthermore, the success rate of the bone grafting procedure and patient satisfaction were evaluated. A visual analogue scale (VAS) was used to determine: A) the degree of pain, with the endpoints of ‘no pain’ (VAS 1) and ‘maximum pain’ (VAS 10); and B) the presence of nerve injury with ‘no sensory disorder’ (VAS 1) and ‘absolute deafness’ (VAS 10). 

Subsequently, patients were followed-up in terms of clinical status and radiological imaging. Radiographs and photographs were acquired for the documentation and analysis of abnormalities. The two-point discrimination test was performed on patients who developed nerve injury to record any irreversible nerve lesion. 

This study was approved by the Ethics Committee of the Faculty of Medicine, Justus Liebig University Giessen, under the approval number (201/11).

Data from the questionnaires and the follow-up examinations were anonymised and coded in an Excel spreadsheet (Excel 2007; Microsoft Corp., Redmond, WA, USA). The data were descriptive, and were analysed using SPSS software (IBM SPSS Statistics, version 22; IBM Corp., Armonk, NY, USA). 

## 3. Results

**Figure 2:**
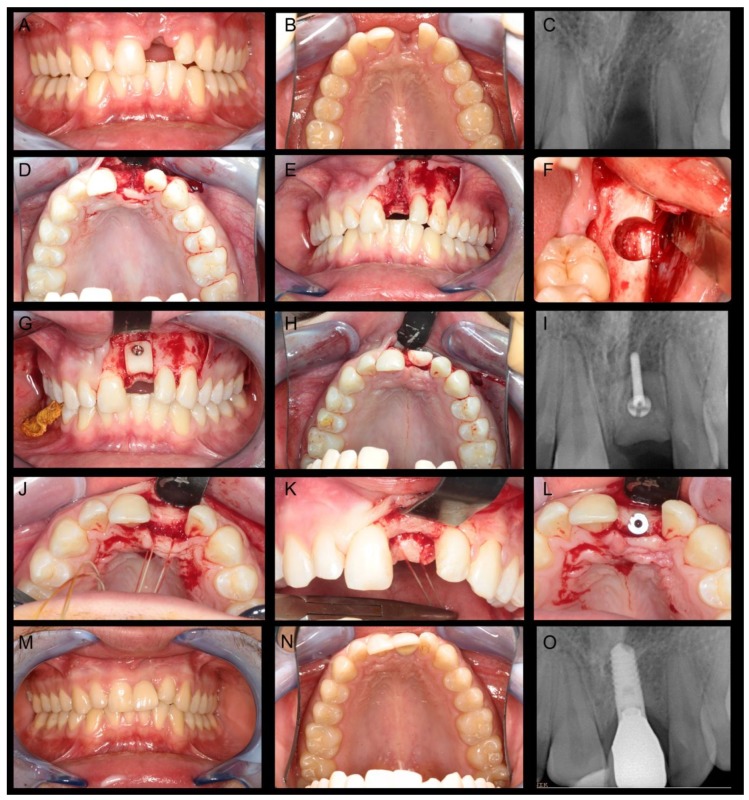
Clinical case report of patient with bone atrophy in the left maxillary central incisor region: (**A**–**C**) Intraoral photos and radiograph show the initial situation; (**D**,**E**) intraoperative photos shows bone defect; (**F**) intraoperative photos after the removal of the standardised bone graft from the left retromolar area of the mandible; (**G**–**I**) Intraoperative photos and radiograph after bone graft fixation using micro-lag-screw; (**J**–**L**) Intraoperative photos 3 month show the bone healing and implant insertion; and (**M**–**O**) intraoral photos and radiograph after prosthetic rehabilitation using ceramic crown.

In a period of almost 13 years, a total of 54 patients with 64 bone cylinders were surgically treated with the here described standardised bone transfer. Fifty-two patients completed the questionnaire, and 48 (92.3%) agreed to clinical follow-up consultations, where 49 bone transplantation sites could be observed. Figure 2 outlines the clinical course of a patient with bone atrophy in the left maxillary central incisor region who was treated with a standardised bone graft from the left retromolar region of the mandible.

For feasible statistical analysis, only the number of bone transplantation were be considered. The pre-therapeutic statistical analysis in this study included data from all treated cases (54 patients, 64 bone cylinders) whereas the follow-up evaluation is based on 48 patients and 49 bone harvesting sites.

**Figure 3:**
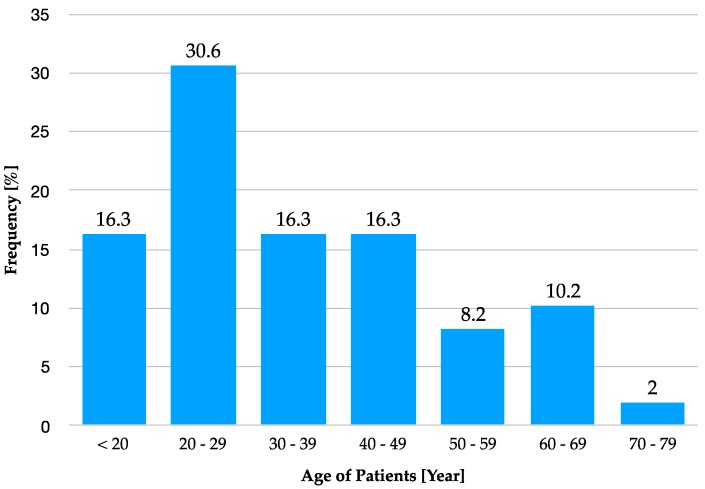
Age distribution of the included patients at the time of the bone graft surgery.

In 49 bone transplantations, 26 procedures (53.1%) were performed in females and 23 (46.9%) in males. The patient age at the time of bone graft surgery ranged between 15 and 75 years (mean 35.18 years) (Figure 3). In one patient, the bone transplant was not successful and the necrotic bone graft with the already inserted dental implant at this region had to be removed. This amounts to a 98% success rate of the transplanted standardised bone graft. 

Forty-six (93.9%) procedures were performed in the outpatient clinic, and three (6.1%) procedures were done in hospitalized patients because additional surgical procedures were performed. The most frequent indication for press fit bone cylinder was alveolar crest atrophy in a single tooth area (n = 46; 93.9%). Two (4.1%) patients underwent sinus lifting surgeries, and one (2%) patient underwent defect filling after a cystectomy. 

**Figure 4:**
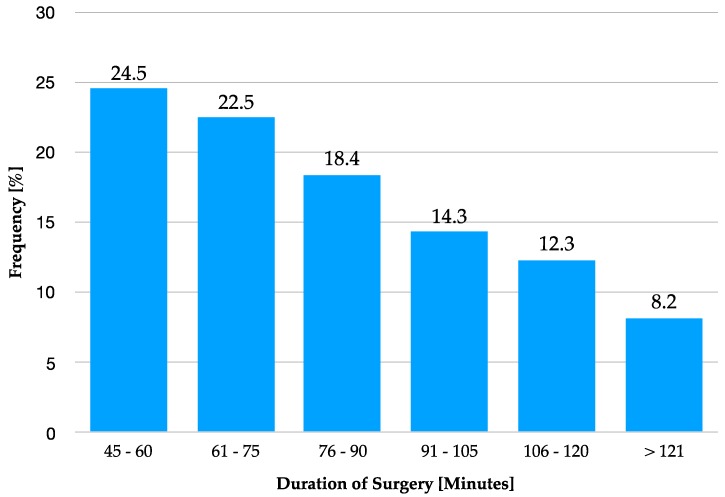
The frequency of the surgery time in minutes.

The average duration of surgeries was 83.7 (± 27.06) min. The shortest procedure lasted 46 min, the longest 153 min. Nearly two-thirds of interventions (65.4%) lasted less than 90 min (see Figure 4).

The surgical bone harvesting was carried out by a total of ten different surgeons, three of them made 71.4% of all interventions. The bone harvesting was performed predominantly from the right mandibular angel (n = 31, 63.3%). In 18 cases (36.7%), the left side was selected.

The average length of the harvested bone cylinders was 8.69 mm. A total of 60.9% of bone transplants were used to augment the upper jaw, and 39.1% were placed in the lower jaw. Almost half (49.9%) of all harvested bone cylinders were transplanted into the anterior teeth region of the upper jaw (Table 1). 

### 3.1. Intraoperative Antibiotic

In 37 procedures (75.5%), patients received an intravenous single-dose antibiotic prophylaxis intraoperatively. Sultamicillin was used in most cases (n = 30, 81.1%). Clindamycin was administered in five cases (13.5%), in two interventions (5.4%), the drug used could no longer be determined. The remaining 12 cases (24.5%) did not receive intraoperative antibiotics.

### 3.2. Complications

**Figure 5:**
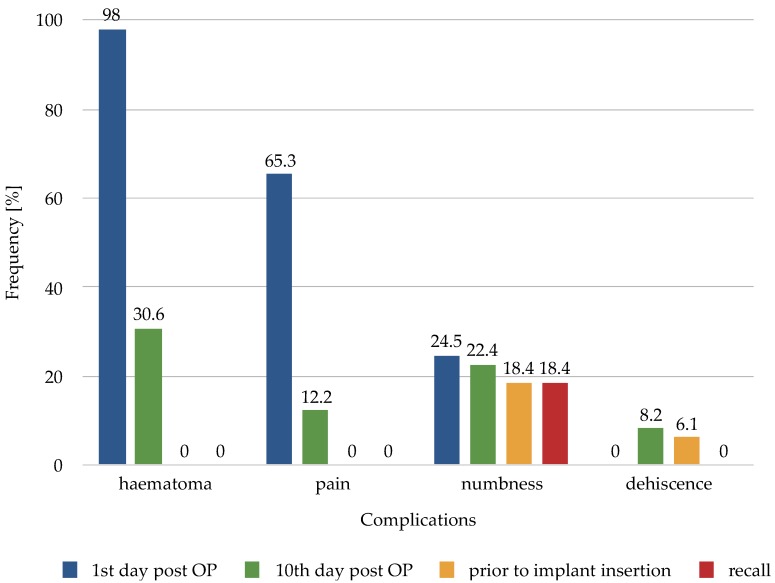
Postoperative complications at different time points.

Intraoperative complications were described in two cases. The mandibular canal was opened after bone harvesting in both procedures. Postoperative complications were recorded at four different time points: one and ten days after surgery, before implant insertion, and at the follow-up examination. At the time of the first postsurgical follow-up (one day after surgery): No patient had experienced wound infection, wound dehiscence, fracture or postoperative bleeding. However, 48 (98%) procedures resulted in haematomas of the buccal mucosa. Thirty-two patients (65.3%) reported pain episodes. Hypoesthesia of the inferior alveolar nerve at the operation site was reported in 12 (24.5%) cases. Ten days after surgery: Wound dehiscence was reported 10 days postoperatively, at the time of suture removal, in four (8.2%) cases. Haematomas were present in 15 (30.6%) cases. Hypoesthesia in the operative area was reported in 11 (22.4%) cases. Pain episodes at this point were reported in six (12.2%) cases. Pre–implant insertion: Three (6.1%) cases still had wound dehiscence at the pre–implant insertion surgical site about three months later, and nine (18.4%) patients reported hypoesthesia. Follow-up examinations: Nine (18.4%) patients reported locally limited hypoesthesia, at the follow-up examinations. No other complication (wound dehiscence, pain episode, or fracture) was reported. The complications recorded at the various postoperative time points are summarised in Figure 5.

On follow-up radiographic examination, the harvest area on the mandibular angle remained visible after 2.5 years in one (2.0%) patient. Delayed re-ossification was seen in the retromolar region due to lower bone density. No radiological abnormality was seen in the rest of the patients (98%). The harvested areas were compared to the pre-surgical sites and were not elevated, lowered, or reduced in density. The intraoral mucosal scar in the operated area was not irritated in any patient.

### 3.3. Patients Related Parameters:

#### 3.3.1. Pain

Postsurgical pain in the harvest area was reported in 37 (69.8%) cases and denied in 16 (30.2%) cases. On average, the patients reported a pain intensity of 4.67 (2.17) of 10 points on the VAS. Of those patients who reported postsurgical pain episodes, 80% were pain free within one week.

#### 3.3.2. Hypoesthesia

Hypoesthesia in the chin region as a result of injury to the inferior alveolar nerve was reported in six (11.3%) cases. Hypoesthesia at the chin persisted in two cases. 

After the conversion of patients’ VAS responses to corresponding numerical values, we concluded that the objectification of disturbance magnitude was difficult.

#### 3.3.3. Functional limitations 

The various functional limitations documented are presented in Table 2. 

#### 3.3.4. Patient satisfaction

More than 94.0% of the patients were very satisfied or satisfied with the general outcome of bone transplantation from the mandibular angle using the Osseo Transfer technique. The average score was 1.58 points (1= very satisfied, 5 = completely unsatisfied). Most (94.2%) patients indicated that they would recommend this procedure in a completely unreserved or reserved way to other patients with similar clinical and surgical indications. 

### 3.4. Summary of Results

In summary, no postsurgical complication occurred in 31 (63.3%) of the 49 bone transplantations from the mandibular angle. In the remaining 18 (36.7%) cases, the documented complications (particularly the neurological problems) need to be differentiated between (1) problems that arose due to the use of the Osseo Transfer technique, and (2) those that were independent of the bone transfer method. The latter problems may have existed prior to the transfer or may have developed after bone rebuilding due to other factors. Table 3 shows the documented complications and their causal relationships to the Osseo Transfer surgery.

## 4. Discussion

This study designed to be a retrospective, clinical, observational study. Due to the fact that no other comparable procedure for bone harvesting was applied in the same hospital, direct comparison of this standardised bone transfer to a control group was not possible. Comparison with another method of operation seems hardly possible. The procedure presented here is performed with a standardised set of instruments and a certain surgical protocol followed common practice to obtain bone through sawing, milling, and chiselling which fit into the size of the anatomical defect. The advantage of the standardised bone transfer is that the recipient area is prepared to fit to the graft, not vice versa as with classical augmentation procedures [11]. Therefore, a direct comparison of this standardised method with other non-standardised methods is not meaningful.

One of the limitations of this study is the subjective assessment of some data which was collected after their occurrence. Patients have different memory skills which can lead to deviations in the information such as pain and its strength. Such data should, therefore, be viewed with caution. This process is also called "recall bias" and should always be considered in the interpretation of results [12].

Surgical procedures in the cranio-maxillofacial field always carry a risk of nerve injury complications [13]. The inferior alveolar nerve is the most affected nerve during bone harvesting from the retromolar area of the mandible [14]. Drilling to an extended depth into the retromolar region may expose the mandibular canal. Partial damage or complete transection may follow. Clinical symptoms include reduced sensibility if the nerve is partially damaged, and complete anaesthesia if it is fully severed. However, the symptoms can range from dysesthesia to hyperesthesia [15,16]. The seriousness of this complication and the need to open the mandibular canal during surgery in two of our cases emphasise the importance of pre-surgical radiological diagnostics to estimate the maximum possible drilling depth above the mandibular nerve canal. Basic diagnostic modalities (i.e., orthopantomography) should be complemented by cone-beam computed tomography in critical borderline cases [17].

In this study, no significant complication was recorded for 31 (63.3%) of the 49 bone transplantations from the retromolar region. In the remaining 18 (36.7%) cases, the sources of documented complications (particularly neurological; i.e. related to or independent of the surgical procedure) need to be differentiated accurately. In three (6.1%) cases, the documented nerve injuries were independent of bone transplantation. Fifteen (30.6%) postoperative complications were related directly to the surgical procedure. Amongst those 15 complications, seven (14.3%) complications were temporary and improved over time, with complete rehabilitation. These observations correspond to reports in the literature that hypoesthesia improves over time, and ultimately can disappear completely [18,19,20]. Persistent manifestation occurred in eight (16.3%) patients, six (12.2%) of them described small, local sensibility deficits.

Another complication can occur when the graft does not properly adapted to the receipt site. This may lead to reduced perfusion of the graft site and therefore to the loss of graft [21]. Success of bone graft significantly depends on the close and tight contact between graft and recipient region [22,23]. Faupel et al. found a complete bony connection between graft and receipt site without formation of intermediate soft tissue in areas of high contact pressure. In areas with lower pressure, connective tissue between graft and bearing bone was formed [24]. In extreme cases, this could lead to sequester formation and consequently to graft loss. The proper use of the Osseo Transfer System produces a press fit junction between original bone and graft preventing such complications [11].

Almost all (97.0%–100%) functional restrictions were temporary. In general, the problems lasted for a few days and no more than a few months.

The patients described postsurgical pain as comparable to pain after other oral surgical procedures in the dento-alveolar area (e.g., extraction of wisdom teeth). This result corresponds to the observations of other authors [25].

Lower bone density in the retromolar region was detected during the clinical follow up examination in only one patient after 2.5 years. The reduced density was a result of delayed re-ossification at the bone cylinder harvest site and might be related to the patient’s advanced age (70 years). However, as the patient was completely free of symptoms, no clinical relevance was attributed to this finding. Jensen and Sindet-Pedersen et al. made a similar observation. They described delayed bone regeneration at harvest sites in patients aged > 60 years at one year after bone transfer surgery [26]. None of the remaining patients in this study had a radiologic abnormality. This finding concurs with the results of Misch et al. and Nkenke et al., who described complete regeneration of retromolar bone harvest sites six months postoperatively [9,27]. Refilling of the bone cavities with artificial bone substitute after bone transfer was not necessary, although other authors have recommended this approach [28]. The retromolar region of the mandible where the bone grafts are harvested consists of very solid bone which is strengthened by trajectory lines derived from the main mastication forces. According to the common understanding of bone healing, functional remodelling takes place and allows for quick and effective healing after removal of limited cylindrical bone grafts [29].

As all donor regions examined in this study were intraoral, no functionally or aesthetically relevant scarring was visible, which significantly increased patient satisfaction. Other authors have also described this advantage of intraoral bone harvesting [9,30].

Application of the Osseo Transfer System is not limited to bone harvesting from the mandibular angle or augmentation of the alveolar bone. The procedure can be used wherever bone regeneration after customised transplantation is desired. Limiting factors are the size of the area that needs to be regenerated and the availability of bone in the donor region.

## 5. Conclusions

Standardised press fit bone cylinder using the Osseo Transfer System surgical kit is a safe, practical, and promising option for the treatment of small and medium-sized alveolar ridge bone defects. This procedure presents good feasibility for the surgeon and impose little stress on patients. Standardised bone transfer surgery has a low rate of complications and is considered as a reliable procedure for patients and surgeons. This method should be applied whenever suitable indications exist.

## Figures and Tables

**Table 1:** Recipient regions (FDI) of bone grafts (frequencies in percent).

-	-	1.6	-	-	1.6	7.8	18.6	17.2	3.1	1.6	6.3	1.6	1.6	-	-
**18**	**17**	**16**	**15**	**14**	**13**	**12**	**11**	**21**	**22**	**23**	**24**	**25**	**26**	**27**	**28**
**48**	**47**	**46**	**45**	**44**	**43**	**42**	**41**	**31**	**32**	**33**	**34**	**35**	**36**	**37**	**38**
-	-	7.8	12.5	-	3.1	1.6	3.1	3.1	-	-	1.6	4.7	1.6	-	-

**Table 2:** Functional limitations.

Functional limitations	NO	YES (total)	YES (temporary < 3 months)	YES (permanent)
**Chewing**	35.8	64.2	62.3	1.9 *
**Speech**	90.6	9.4	9.4	-
**Swallowing**	96.2	3.8	3.8	-
**Dysgeusia**	98.1	1.9	1.9	-
**Mouth opening**	37.7	62.3 **	60.4	-

* Consequences of a trauma; independent of standardised Osseo Transfer. ** One patient stated that the functional restriction at mouth opening lasted six months.

**Table 3:** Summary of postoperative complications.

Complications [post OP]	Frequency [n]	Frequency [%]
No abnormality	31	63.3
Temporary abnormality,	7	14.3
Osseo Transfer **(causal)**		
Permanent abnormality,	8	16.3
Osseo Transfer **(causal)**		
Abnormality, Osseo Transfer **(not causal)**	3	6.1
